# Characterisation of Shiga toxin-producing *Escherichia coli *O157 strains isolated from humans in Argentina, Australia and New Zealand

**DOI:** 10.1186/1471-2180-8-46

**Published:** 2008-03-17

**Authors:** Gerardo A Leotta, Elizabeth S Miliwebsky, Isabel Chinen, Estela M Espinosa, Kristy Azzopardi, Sharon M Tennant, Roy M Robins-Browne, Marta Rivas

**Affiliations:** 1 Servicio Fisiopatogenia, Instituto Nacional de Enfermedades Infecciosas – ANLIS "Dr. Carlos G. Malbrán". Av. Vélez Sarsfield 563 (1281) Ciudad Autónoma de Buenos Aires, Argentina; 2 Consejo Nacional de Investigaciones Científicas y Técnicas (CONICET), Argentina; 3 Department of Microbiology and Immunology, University of Melbourne, and Murdoch Childrens Research Institute, Royal Children's Hospital, Parkville Victoria 3010, Australia

## Abstract

**Background:**

Shiga toxin-producing *Escherichia coli *(STEC) is an important cause of bloody diarrhoea (BD), non-bloody diarrhoea (NBD) and the haemolytic uraemic syndrome (HUS). In Argentina and New Zealand, the most prevalent STEC serotype is O157:H7, which is responsible for the majority of HUS cases. In Australia, on the other hand, STEC O157:H7 is associated with a minority of HUS cases. The main aims of this study were to compare the phenotypic and genotypic characteristics of STEC O157 strains isolated between 1993 and 1996 from humans in Argentina, Australia and New Zealand, and to establish their clonal relatedness.

**Results:**

Seventy-three O157 STEC strains, isolated from HUS (n = 36), BD (n = 20), NBD (n = 10), or unspecified conditions (n = 7) in Argentina, Australia and New Zealand, were analysed. The strains were confirmed to be *E. coli *O157 by biochemical tests and serotyping. A multiplex polymerase chain reaction (PCR) was used to amplify the *stx*_1_, *stx*_2 _and *rfb*_O157 _genes and a genotyping method based on PCR-RFLP was used to determine *stx*_1 _and *stx*_2 _variants. This analysis revealed that the most frequent *stx *genotypes were *stx*_2_/*stx*_2c (vh-a) _(91%) in Argentina, *stx*_2 _(89%) in New Zealand, and *stx*_1_/*stx*_2 _(30%) in Australia. No *stx*_1_-postive strains were identified in Argentina or New Zealand. All strains harboured the *eae *gene and 72 strains produced enterohaemolysin (EHEC-Hly). The clonal relatedness of strains was investigated by phage typing and pulsed-field gel electrophoresis (PFGE). The most frequent phage types (PT) identified in Argentinian, Australian, and New Zealand strains were PT49 (n = 12), PT14 (n = 9), and PT2 (n = 15), respectively. Forty-six different patterns were obtained by *Xba*I-PFGE; 37 strains were grouped in 10 clusters and 36 strains showed unique patterns. Most clusters could be further subdivided by *Bln*I-PFGE.

**Conclusion:**

STEC O157 strains isolated in Argentina, Australia, and New Zealand differed from each other in terms of *stx*-genotype and phage type. Additionally, no common PFGE patterns were found in strains isolated in the three countries. International collaborative studies of the type reported here are needed to detect and monitor potentially hypervirulent STEC clones.

## Background

Shiga toxin-producing *Escherichia coli *(STEC) is an important emerging pathogen which can cause bloody diarrhoea (BD), non-bloody diarrhoea (NBD) and the haemolytic uraemic syndrome (HUS). The ability of STEC strains to cause severe disease in humans is related to their capacity to secrete Shiga toxins, Stx1 and Stx2, and variants of these toxins [1,2]. Another virulence-associated factor of most STEC isolates associated with severe disease is intimin, a 94-kDa outer membrane protein, which is encoded by the *eae *gene on a ca. 34-kb chromosomal pathogenicity island termed the locus of enterocyte effacement (LEE). This locus is associated with the intimate adherence of *E. coli *to epithelial cells, initiation of host signal transduction pathways, and the formation of attaching-and-effacing intestinal lesions [3]. In addition, most STEC strains associated with BD or HUS produce an enterohaemolysin (EHEC-Hly), encoded by a plasmid-borne gene, known as *ehx*A [4].

Argentina has a high incidence of HUS: 13.9 cases per 100,000 children younger than 5 years old were reported in 2005 [5]. This rate is 10-fold higher than in other industrialised countries [6]. In Argentina STEC is the primary aetiological agent of HUS, and *E. coli *O157:H7 is the predominant serotype isolated [7]. In Australia and New Zealand the annual incidence of HUS, determined through active surveillance, is approximately 1.0 to 1.3 per 100,000 children less than 5 years old [8]. Interestingly, the predominant STEC serotypes associated with HUS in these two countries differs. Whereas in New Zealand O157 strains make up around half of the isolates, in Australia serotype O111 STEC account for most HUS cases, with O157 associated with fewer than 20% [8,9]. The reasons for these differences are not known.

The aims of this study were to compare the phenotypic and genotypic characteristics of STEC O157 strains isolated from humans in Argentina, Australia and New Zealand during the period 1993–1996, and to establish the genetic diversity and clonal relatedness among the strains isolated in these three countries. The rationale for undertaking this study was based upon national and international concerns that modern centralised and rapid food distribution systems in these countries together with recent increases in the volume of food trade internationally and the enormous increase in global travel could allow the unfettered, and undetected, worldwide spread of virulent clones of STEC O157.

## Results

Of the 73 strains examined (35 from Argentina, 20 from Australia and 18 from New Zealand), 36 were isolated from HUS cases, 20 from BD cases, and 10 from NBD cases. The clinical origin of 7 strains was not specified (Table 1). All strains were confirmed as *E. coli *O157, were cytotoxic for Vero cells, and carried the genes encoding intimin and *fli*C_H7_, even though several were non-motile in vitro, and were typed as O157:H- (data not shown). Production of enterohaemolysin was observed in 72 (98.6%) of 73 strains, in agreement with the polymerase chain reaction (PCR) results.

**Table 1:** Clinical presentation of patients in Argentina, Australia, and New Zealand from whom STEC O157 strains were isolated between 1993 and 1996.

	No. (%) of strains from:		
	
Clinical presentation	Argentina	Australia	New Zealand
Haemolytic uraemic syndrome	22 (63)	6 (30)	8 (44)
Bloody diarrhoea	10 (29)	3 (15)	7 (39)
Non-bloody diarrhoea	3 (9)	5 (25)	2 (22)
Non-specific	0 (0)	6 (30)	1 (6)

Total	35 (100)	20 (100)	18 (100)

PCR-restriction fragment length polymorphism (RFLP) genotyping of the 73 O157 STEC strains showed that 35 (48%) strains harboured *stx*_2 _and *stx*_2c (vh-a)_, 22 (30%) carried only *stx*_2_, 6 (8%) carried *stx*_1 _and *stx*_2_, 4 (5%) carried *stx*_1 _and *stx*_2c (vh-a)_, 3 (4%) carried only *stx*_2c (vh-a)_, 2 (3%) carried only *stx*_1 _and 1 (1%) carried *stx*_1 _and *stx*_2-UT _(Table 2). The most frequent *stx *genotypes were *stx*_2 _and *stx*_2c (vh-a) _(32/35; 91%) in Argentina, *stx*_1 _and *stx*_2 _(6/20; 30%) in Australia, and *stx*_2_(16/18; 89%) in New Zealand. *stx*_2 _was the only *stx*-genotype found in all 3 countries. No *stx*_1_-positive strains were obtained from patients in Argentina or New Zealand, compared with 13 of 20 from Australia (*P *< 0.0001; Fisher's exact test, two-tailed). Of the 13 *stx*_1_-positive strains isolated in Australia, however, 11 were also positive for stx_2 _or *stx*_2c_. Notwithstanding these difference in toxin profile, there was no significant association between *stx *genotype and the clinical presentation of the patient from whom the strain was obtained (Table 3; *P *> 0.5, Kruskal-Wallis test).

**Table 2:** *stx *genotype of STEC O157 strains isolated between 1993 and 1996 from humans in Argentina, Australia and New Zealand.

	No. (%) of strains from:		
	
*stx *genotype	Argentina	Australia	New Zealand
*stx*_1_	0	2 (10)	0
*stx*_2_	3 (9)	3 (15)	16 (89)
*stx*_2c (vh-a)_	0	1 (5)	2 (11)
*stx*_1_/*stx*_2_	0	6 (30)	0
*stx*_1_/*stx*_2c (vh-a)_	0	4 (20)	0
*stx*_1_/*stx*_2-UT_	0	1 (5)	0
*stx*_2_/*stx*_2c (vh-a)_	32 (91)	3 (15)	0

Total	35 (100)	20 (100)	18 (100)

**Table 3:** Distribution of STEC O157 strains isolated from humans with haemolytic uraemic syndrome (HUS), bloody diarrhoea (BD), non-bloody diarrhoea (NBD) or other non-specified conditions (NS) according to *stx *genotype.

	No. (%) of strains from patients with :			
	
*stx *genotype	HUS	BD	NBD	NS
*stx*_1_	0	0	2 (20)	0
*stx*_2_	12 (33)	6 (30)	3 (30)	1 (14)
*stx*_2c (vh-a)_	0	2 (10)	1 (10)	0
*stx*_1_/*stx*_2_	1 (3)	0	0	5 (71)
*stx*_1_/*stx*_2c (vh-a)_	1 (3)	2 (10)	1 (10)	0
*stx*_1_/*stx*_2-UT_	0	0	0	1 (14)
*stx*_2_/*stx*_2c (vh-a)_	22 (61)	10 (50)	3 (30)	0

Total	36 (100)	20 (100)	10 (100)	7 (100)

The most frequent phage types (PT) identified in Argentina, Australia, and New Zealand were PT49 (12/35; 34%), PT14 (9/20; 45%), and PT2 (15/18; 83%), respectively (Table 4). PT4 was the only PT found in all 3 countries with frequencies of 31% (11/35) in Argentina; 15% (3/20) in Australia, and 11% (2/18) in New Zealand. There was no significant association between phage type and the clinical presentation of the patient from whom the strain was obtained (*P *> 0.5, Kruskal-Wallis test).

**Table 4:** Phage type of STEC O157 strains isolated between 1993 and 1996 from humans in Argentina, Australia and New Zealand.

	No. (%) of strains from:		
	
Phage type	Argentina	Australia	New Zealand
1	0	1 (5)	0
2	8 (23)	0	15 (83)
4	11 (31)	3 (15)	2 (11)
14	1 (3)	9 (45)	0
21	0	1 (5)	0
26	2 (6)	1 (5)	0
32	0	0	1 (6)
49	12 (34)	0	0
54	0	3 (15)	0
Untypeable	1 (3)	2 (10)	0

Total	35 (100)	20 (100)	18 (100)

Pulsed-field gel electrophoresis (PFGE) of *Xba*I-digested genomic DNA was used to subtype all 73 STEC O157 strains, generating 46 distinct PFGE patterns with 16–24 discernible fragments, ranging from approximately 32 to 730 kb in size. Twenty-one patterns were identified amongst 35 strains isolated in Argentina, 14 patterns in 20 strains from Australia, and 11 patterns in 18 strains from New Zealand.

**Figure 1:**
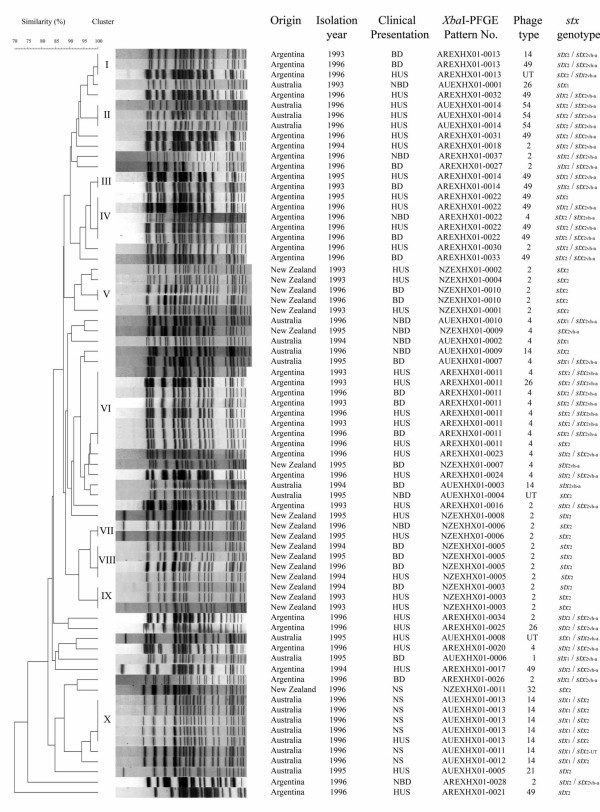
Clonal relationship of 73 STEC O157 strains isolated between 1993 and 1996 from humans in Argentina, Australia, and New Zealand.

Ten clusters contained isolates with indistinguishable *Xba*I-PFGE profiles (Figure 1). These were cluster I (3 strains from Argentina); cluster II (3 strains from Australia); cluster III (2 strains from Argentina); cluster IV (5 strains from Argentina); cluster V (2 strains from New Zealand); cluster VI (8 strains from Argentina); cluster VII (2 strains from New Zealand); cluster VIII (4 strains from New Zealand); cluster IX (3 strains from New Zealand); and cluster X (5 strains from Australia). To refine the cluster analysis further, we determined the *Bln*I-PFGE profile of each isolate. This revealed that most strains with identical *Xba*I-PFGE pattern could be discerned from each other by restriction with a second enzyme (Table 5). Some clusters could also be split by using phage typing and *stx *genotyping (Table 5). Only two clusters, II (3 strains) and X (5 strains), both from Australia, were indistinguishable by *XbaI*- and *BlnI*-PFGE, phage typing and *stx*-genotyping, indicating that these were probable clones.

**Table 5:** Discrimination of STEC O157 strains within *Xba*I-PFGE clusters by phage type, *stx *genotype and *Bln*I-PFGE.

Cluster/No. of *Xba*I-PFGE	No. of strains	Phage type	*stx *genotype	*Bln*I-PFGE pattern
I (AREXHX01-0013)	1	14	*stx*_2 _and *stx*_2c (vh-a)_	AREXHA26-0013
	1	49	*stx*_2 _and *stx*_2c (vh-a)_	AREXHA26-0015
	1	UT	*stx*_2 _and *stx*_2c (vh-a)_	AREXHA26-0024
II (AUEXHX01-0014)	3	54	*stx*_2 _and *stx*_2c (vh-a)_	AUEXHA26-0002
III (AREXHX01-0014)	1	49	*stx*_2 _and *stx*_2c (vh-a)_	AREXHA26-0018
	1	49	*stx*_2 _and *stx*_2c (vh-a)_	AREXHA26-0015
IV (AREXHX01-0022)	3	49	*stx*_2 _and *stx*_2c (vh-a)_	AREXHA26-0015
	1	4	*stx*_2 _and *stx*_2c (vh-a)_	AREXHA26-0015
	1	49	*stx*_2_	AREXHA26-0019
V (NZEXHX01-0010)	1	2	*stx*_2_	NZEXHA26-0003
	1	2	*stx*_2_	NZEXHA26-0007
VI (AREXHX01-0011)	2	4	*stx*_2 _and *stx*_2c (vh-a)_	AREXHA26-0008
	1	4	*stx*_2 _and *stx*_2c (vh-a)_	AREXHA26-0031
	1	26	*stx*_2 _and *stx*_2c (vh-a)_	AREXHA26-0011
	1	4	*stx*_2 _and *stx*_2c (vh-a)_	AREXHA26-0023
	1	4	*stx*_2 _and *stx*_2c (vh-a)_	AREXHA26-0022
	1	4	*stx*_2 _and *stx*_2c (vh-a)_	AREXHA26-0009
	1	4	*stx*_2_	AREXHA26-0025
VII (NZEXHX01-0006)	1	2	*stx*_2_	NZEXHA26-0005
	1	2	*stx*_2_	NZEXHA26-0004
VIII (NZEXHX01-0005)	2	2	*stx*_2_	NZEXHA26-0001
	1	2	*stx*_2_	NZEXHA26-0002
	1	2	*stx*_2_	NZEXHA26-0006
IX (NZEXHX01-0003)	1	2	*stx*_2_	NZEXHA26-0001
	1	2	*stx*_2_	NZEXHA26-0002
	1	2	*stx*_2_	NZEXHA26-0003
X (AUEXHX01-0013)	5	14	*stx*_1 _and *stx*_2_	AUEXHA26-0001

In Argentina, the predominant *Xba*I-PFGE patterns were AREXHX01-0011 (cluster VI, 8 strains), AREXHX01-0022 (cluster IV, 5 strains), AREXHX01-0013 (cluster I, 3 strains) and AREXHX01-0014 (cluster III, strains). The eight strains in cluster VI were recovered from three HUS cases and one BD case in 1993. and from two HUS cases and two BD cases in 1996. All of these isolates carried *stx*_2 _and *stx*_2c (vh-a)_, except one strain isolated from a patient with HUS which carried *stx*_2 _only. Seven strains belonged to PT4, and one to PT26. Four of the five strains in cluster IV carried *stx*_2 _and *stx*_2c (vh-a)_. Four strains in this cluster were isolated from one case each of NBD, BD, and two of HUS in 1996. Three of these strains belonged to PT49, and one to PT4. The three *stx*_2_/*stx*_2c (vh-a) _PT49 strains in this cluster showed the same pattern on *Bln*I-PFGE and are likely to be a clone. The remaining strain in this cluster carried *stx*_2_, belonged to PT49, and was recovered from an HUS case in 1995. The three strains in cluster I carried *stx*_2 _and *stx*_2c (vh-a) _and were isolated from a BD case in 1993, and from a BD and a HUS case in 1996. All three belonged to different PTs (PT49, PT14, and PT-UT). The two strains in cluster III, carried *stx*_2 _and *stx*_2c (vh-a)_, belonged to PT49 and were isolated from a BD case in 1993, and an HUS case in 1995.

In Australia, the predominant *Xba*I-PFGE patterns were AUEXHX01-0013 (cluster X, 5 strains), and AUEXHX01-0014 (cluster II, 3 strains). All five strains in cluster X carried *stx*_1 _and *stx*_2_, belonged to PT14, and were isolated in 1996 from one HUS case, two contacts of this case and from two individuals with an unspecified disease other than HUS or diarrhoea. These strains also showed the same *Bln*I-PFGE pattern (Table 5). All three strains in cluster II carried *stx*_2 _and *stx*_2c (vh-a)_, belonged to PT54 and were isolated from HUS cases in 1996. The strains were also indistinguishable by *Bln*I-PFGE. The only enterohaemolysin-negative strain in this study carried *stx*_2 _alone, belonged to PT21 and was isolated from a HUS case in 1995.

In New Zealand, the predominant *Xba*I-PFGE patterns were NZEXHX01-0005 (cluster VIII, 4 strains), NZEXHX01-0003 (cluster IX, 3 strains), NZEXHX01-0010 (cluster V, 2 strains), and NZEXHX01-0006 (cluster VII, 2 strains). The four strains in cluster VIII were isolated from BD cases in 1994, 1995 and 1996, and from a HUS case in 1994. The three strains in cluster IX were obtained from two HUS cases in 1993 and one case of BD in 1994. The two strains in cluster V were isolated from BD cases in 1996, and the two strains in cluster VII were isolated from an HUS case in 1995 and a NBD case in 1996. All 11 strains harboured *stx*_2 _and were the same phage type (PT2).

## Discussion

This study revealed differences in the *stx *genotype, phage type and PFGE profiles of clinically-significant STEC O157 strains isolated from humans in Argentina, Australia and New Zealand. Strains that possessed both *stx*_1 _and *stx*_2 _were relatively more prevalent in Australia as is the case in the USA [10], Japan [11] and Chile [12], amongst others. On the other hand, the relatively high prevalence of *stx*_2_-positive strains in New Zealand resembles the toxin profile of STEC O157 in Western Europe [13,14]. In Argentina, all isolates were either *stx*_2 _or and *stx*_2_/*stx*_2c (vh-a)_, with *stx*_2_/*stx*_2c (vh-a) _strains accounting for more than 90% of isolates. This highly virulent genotype has been described by Nishikawa *et al*. [15] as being associated with severe human disease. The high frequency of this toxin profile in isolates from Argentina, and that of Stx2-producing STEC in New Zealand, which are also reportedly more virulent than other strains [16], compared with Australia (Table 2), may account in part for the relatively low frequency of severe infection with STEC O157 in Australia compared with the other countries. Our finding that *stx *profile was not significantly associated with clinical presentation, may have been due to a sampling error, in that isolates from the sickest patients are more likely to be sent to reference laboratories for characterisation.

At least 90 phage types have been reported for STEC O157 [17], but only four of these (PT2, PT4, PT14, and PT49) accounted for 84% of the strains analysed in this study. In Europe and Canada, PT2, PT4, PT8, PT14, PT21, PT32, and PT54 account for the majority (> 75%) of STEC O157 strains obtained from humans [18]. Six of these PTs (PT2, PT4, PT14, PT21, PT32, and PT54), were identified in the present study, but only PT4 occurred in all three countries. PT2 and PT8 predominate among human strains of STEC O157 in Spain, Belgium, Finland, Germany, Italy, England and Scotland [17,19]. In our study, PT2 was predominant in New Zealand strains, and also was identified among the Argentinian strains, but not in Australian strains. PT8 was not identified in this study. PT14, predominant in Canada [17], was identified in Argentinian and Australian strains, but not in those from New Zealand. PT49 and PT4 were common in Argentina, but absent (PT49) or infrequent (PT4) in Australia and New Zealand.

PFGE is the ''gold standard'' of genetic fingerprinting methods for *E. coli *O157 and is particularly useful for tracking outbreak strains [20]. In this study a high degree of diversity was observed among the strains analysed by this technique. No common *Xba*I-PFGE pattern was found in strains isolated in Argentina, Australia or New Zealand. However, different clusters were detected in each country. The predominant *Xba*I-PFGE patterns were: AREXHX01-0011 (8 strains) in Argentina, AUEXHX01-0013 (5 strains) in Australia and NZEXHX01-0005 (4 strains) in New Zealand. The EXHX01-0011 and EXHX01-0022 patterns that were frequent in this study are common within the Argentinian Database, comprising 30% of the STEC O157 isolates detected in different regions of Argentina during the last 10 years [5]. AREXHX01-0011 is indistinguishable from EXHX01-0047, the second most common pattern in the US national database, accounting for approximately 4.6% of strains. The most recent multi-state outbreak with a strain of this pattern occurred in 2003 and was linked to beef, resulting in a food recall [21].

All but two of the *Xba*I-PFGE clusters could be subdivided by one or more of *stx *genotyping, phage typing and *Bln*I-PFGE (Table 5). Those which could not be subdivided in this way included three strains that were epidemiologically related, and others that may have been responsible for small, undetected community outbreaks of infection. In the absence of pertinent data about the patients from whom these strains were obtained, however, we were unable to test this hypothesis.

## Conclusion

The 73 STEC O157 strains isolated between 1993 and 1996 from symptomatic humans in Argentina, Australia and New Zealand differed in terms of *stx*-genotype and phage type. No common *Xba*I-PFGE pattern was found in the strains isolated in the three countries, indicating that there up until 1996 there was little spread of STEC O157 between them. Most isolates with common *Xba*I-PFGE patterns could be distinguished form each other by *stx *genotyping, phage typing or *Bln*I-PFGE. By conducting international collaborative studies, using standardised detection and typing tools of the type reported here, reference laboratories will be able to detect the emergence of potentially hypervirulent STEC clones and monitor their spread.

## Methods

### Bacterial strains

All 73 *E. coli *O157 strains of human origin submitted to the relevant microbiology reference laboratories in Argentina, Australia and New Zealand between 1993 and 1996 were studied. Each strain represented a single isolate from a particular patient and none was associated with a recognised outbreak. The 35 Argentinian strains were submitted to the National Reference Laboratory by different hospitals. The 20 Australian and 18 New Zealand strains were submitted by various diagnostic laboratories in both countries to the Department of Microbiology and Immunology, University of Melbourne, for confirmation of their identity and further characterisation. In keeping with the guidelines of our human ethics committees, all strains were coded to protect patient confidentiality. The distribution of strains isolated from patients in Argentina, Australia, and New Zealand by clinical presentation is shown in Table 1.

### Phenotypic and genotypic characteristics of isolates

All strains were confirmed to be *E. coli *O157 by biochemical tests [22], and serotyping using somatic and flagellar antisera obtained from the Instituto Nacional de Producción de Biológicos – ANLIS "Dr. Carlos G. Malbrán" [23]. The presence of *stx*_1_, *stx*_2 _and *rfb*_O157 _was determined by means of a multiplex PCR [24]. The reference *E. coli *strains, EDL933 O157:H7 (*stx*_1_, *stx*_2_), and non-pathogenic *E. coli *ATCC 25922 were used as positive and negative controls, respectively. Enterohaemolysin production was detected on sheep blood agar plates [25]. To determine Stx production, bacterial supernatant and periplasmic cell extracts were used in cytotoxicity assays on Vero cells [26] using Stx1- and Stx2- specific monoclonal antibodies (MAb 13C4 and BC5BB12, respectively) kindly provided by Dr. N. A. Strockbine, Centers for Disease Control and Prevention, Atlanta, GA, USA. Additional virulence markers were characterised by PCR. The intimin (*eae*) and *ehxA *genes were detected by PCR [4,27]. The presence of the *fli*C_H7 _gene was tested as described by Gannon *et al*. [28].

### Subtyping of isolates

Genotyping of *stx*_2 _variants was done by RFLP analysis of the B-subunit-encoding DNA fragments obtained by PCR [29] and extended to include primers and restriction enzymes as described by Pierard *et al*. [30]. The reference *E. coli *strains 92–3580 O157:H7 (*stx*_2vh-a_) and 93–016 O113:H21 (stx_2vh-b_) were kindly provided by Dr. D. Woodward, of the National Microbiology Laboratory, Public Health Agency ofCanada, Winnipeg, Manitoba, Canada. *stx*_1 _variants were identified by RFLP and PCR [31]. Phage typing was performed by the method originally described by Ahmed *et al*. [32] and extended by Khakhria *et al*. [17]. The *E. coli *O157 typing phages used were kindly provided by R. Ahmed, of the National Microbiology Laboratory, Winnipeg, Manitoba, Canada. Macrorestriction fragment analysis by PFGE was performed using the 24-h PulseNet standardised PFGE protocol for *E. coli *O157:H7 [33] with minor modifications. Restriction digestion of DNA embedded in plugs was performed with 30 U of *Xba*I or *Bln*I (Promega Corp., Madison, WI, USA) at 37°C overnight. The PulseNet size standard strain used was *Salmonella *Braenderup H9812 (kindly provided by the Centers for Disease Control, Atlanta, GA, USA). DNA fragments were resolved in 1% pulsed-field SeaKem Gold agarose (FMC Bioproducts, Rockland, ME, USA) in 0.5× TBE buffer at 14°C. PFGE was performed using a CHEF DR-III system (Bio-Rad Laboratories, Hercules, CA, USA), using a linear pulse ramp of 2.2–54.2 sec with a run length of 18 h and a constant voltage of 200 V. Images of gels were obtained using a Gel Doc 2000 (Bio-Rad Laboratories). Analysis of TIFF images was performed using the BioNumerics software package (Applied Maths, Kortrijk, Belgium) using the Dice coefficient and UPGMA to generate dendrograms with 1.5% tolerance values. The clonal relationship analysis was confirmed visually.

### Statistical analysis

Statistical analysis of data was performed using InStat version 3.05 (GraphPad Software Inc., San Diego, CA, USA).

## Authors' contributions

GAL and EME performed the detection of virulence genes by PCR, the typing of the *stx *genes by RFLP-PCR and *Xba*I-PFGE. EM was the responsible for the phage typing and the cytotoxicity assays on Vero cells. GAL and IC carried out the analysis of the PFGE results. MR designed the study and, together with GAL, drafted the manuscript. KA, ST and RRB were involved in the initial isolation and characterisation of the Australian and New Zealand strains. RRB prepared the final version of the manuscript together with GAL and MR. All authors read, commented on and approved the final manuscript.
